# Use of Decellularized Bio-Scaffolds for the Generation of a Porcine Artificial Intestine

**DOI:** 10.3390/mps7050076

**Published:** 2024-09-27

**Authors:** Sharon Arcuri, Georgia Pennarossa, Madhusha Prasadani, Fulvio Gandolfi, Tiziana A. L. Brevini

**Affiliations:** 1 Department of Veterinary Medicine, Università degli Studi di Sassari, Via Vienna, 07100 Sassari, Italy; sarcuri@uniss.it; 2 Laboratory of Biomedical Embryology and Tissue Engineering, Department of Veterinary Medicine and Animal Sciences, Università degli Studi di Milano, Via dell’Università 6, 26900 Lodi, Italy; georgia.pennarossa@unimi.it; 3 Institute of Veterinary Medicine and Animal Sciences, Estonian University of Life Sciences, 50411 Tartu, Estonia; madhusha.gamage@emu.ee; 4 Department of Agricultural and Environmental Sciences—Production, Landscape, Agroenergy, Università degli Studi di Milano, Via Celoria 2, 20133 Milan, Italy; fulvio.gandolfi@unimi.it

**Keywords:** 3D model, decellularization, extracellular matrix, intestine, repopulation

## Abstract

In recent years, great interest has been focused on the development of highly reproducible 3D in vitro models that are able to mimic the physiological architecture and functionality of native tissues. To date, a wide range of techniques have been proposed to recreate an intestinal barrier in vitro, including synthetic scaffolds and hydrogels, as well as complex on-a-chip systems and organoids. Here, we describe a novel protocol for the generation of an artificial intestine based on the creation of decellularized bio-scaffolds and their repopulation with intestinal stromal and epithelial cells. Organs collected at the local slaughterhouse are subjected to a decellularization protocol that includes a freezing/thawing step, followed by sequential incubation in 1% SDS for 12 h, 1% Triton X-100 for 12 h, and 2% deoxycholate for 12 h. At the end of the procedure, the generated bio-scaffolds are repopulated with intestinal fibroblasts and then with epithelial cells. The protocol described here represents a promising and novel strategy to generate an in vitro bioengineered intestine platform able to mimic some of the complex functions of the intestinal barrier, thus constituting a promising 3D strategy for nutritional, pharmaceutical, and toxicological studies.

## 1. Introduction

Until a few decades ago, the most widely used strategy to study the mechanisms underlying cell biology in vitro was represented by two-dimensional (2D) culture systems [1]. The major advantage of such an approach is represented by the simple and low-cost maintenance. However, 2D cultures only partly mimic the physiological environment of the original tissues, thus leading to potential alterations in cell morphology, polarity, differentiation, metabolism proliferation, growth, gene expression pattern, secretion activity, and several other functions [2,3,4]. To overcome these limits, three-dimensional (3D) models have been developed and introduced to maintain the main properties of tissues and encourage cell-to-cell and cell-to-extracellular matrix interactions, controlling cell behaviors and tissue and organ properties [5,6,7].

The several 3D culture supports currently developed are classified based on their synthetic or biological composition. The first includes hydrogels, microbioreactors, transwell inserts, and highly porous scaffolds, which are made of synthetic polymers, such as polyethylene terephthalate (PET), polyacrylamide (PAM), and polyethylene glycol (PEG) [8,9,10,11,12,13,14,15,16]. The second is constituted by biological polymers, namely, collagen, fibrin, chitosan, alginate, and gelatin, and are represented by 3D printed bio-scaffolds and decellularized organs [17,18,19,20,21,22,23,24,25]. The latter is obtained by exploiting both mechanical forces and chemical reagents that remove cellular components while preserving the intact extracellular matrix (ECM) and maintaining its structural, biochemical, and biomechanical properties [5,26,27,28]. This allows for the production of biological scaffolds that, in contrast to other 3D synthetic culture supports, preserve the organ-specific architecture and stiffness, thus encouraging cell distribution and homing and inducing a more physiological cell behavior, including proliferation, differentiation, and function [18,29,30]. In addition, the removal of native cells and genetic materials makes bio-scaffolds promising tools for allotransplantation, thanks to their low immunogenicity and biocompatibility [29,31]. Interestingly, this approach can be applied to any tissue and organ, as demonstrated by several tissue-specific protocols developed for the successful creation of natural bio-scaffolds, such as the kidney [32,33], heart [34], liver [25,35], nerve [30], and cornea [31]. Overall, decellularized scaffolds represent innovative culture platforms that faithfully recapitulate in vitro the highly complex 3D tissue structures distinctive of the original organ. On the other hand, further studies are currently in progress to optimize and scale up this technique to ensure tissue- and organ-specific acellular bio-scaffold derivation for tissue engineering and regenerative medicine.

An interesting application of 3D approaches has been dedicated to the creation of intestinal models for nutritional, nutraceutical, cytotoxicity, and food and drug absorption studies [36]. In this context, the recreation of the intestinal barrier in vitro has been carried out mainly using synthetic scaffolds [11,15]. In particular, the most common strategy involved is the use of transwell inserts, which allows a single epithelial compartment with polarized cells to be generated [37,38,39,40]. The limitation of this technique is the lack of a stromal compartment that is important for physiological cell-to-cell and cell-to-ECM interactions, which, in turn, are known to influence intestinal epithelial structure and function. This has encouraged the development of alternative synthetic cell culture supports based on highly porous scaffolds, which, when repopulated with appropriate cells, allow both the stroma and the epithelial compartments to be recreated in vitro [8,11]. In particular, these polystyrene-based supports display specific porosity that provides a physical space for fibroblasts to infiltrate, maintain their natural shape, and freely interact in 3D, producing/releasing endogenous extracellular matrix proteins. Notably, their thickness of around 200 µm closely mimics the distance of cells from blood capillaries, which represents the cell nutrient source in vivo [16].

All these models are based on synthetic materials, and only more recently, biological scaffolds have been created that maintain both the ECM complex structure and components, encouraging cell-to-cell and cell-to-ECM interactions [41,42,43,44]. 

Here, we describe a protocol that takes advantage of an “*ad hoc*” decellularization procedure to generate an artificial intestinal platform in vitro, which is then repopulated with intestinal fibroblasts and epithelial cells. Specifically, porcine intestines are collected at the local slaughterhouse and immediately transported to the laboratory. Organs are then subjected to a tissue-specific decellularization protocol that includes a freeze/thaw step, followed by sequential incubation in 1% SDS for 12 h, 1% Triton X-100 for 12 h, and 2% deoxycholate for 12 h. In parallel, fibroblasts and epithelial cells are isolated from porcine intestinal biopsies and grown in vitro using appropriate culture media. Lastly, the generated bio-scaffolds are repopulated with intestinal fibroblasts and epithelial cells to generate artificial intestines.

This multi-step approach represents an innovative and promising tool for the generation of bioengineered intestine platforms that mimic the complex architecture and functions of the intestinal barrier, thus constituting a highly predictive in vitro model for nutritional, pharmaceutical, and toxicological studies. 

## 2. Experimental Design

### 2.1. Materials

- Porcine intestine;
- Sterile plastic containers (Sigma-Aldrich, Milan, Italy; Cat. No.: 861197);
- Ice container;
- Dulbecco’s phosphate-buffered saline (PBS) (Sigma-Aldrich, Milan, Italy, Cat. No.: D5652);
- Antibiotic/antimycotic solution (Sigma-Aldrich, Milan, Italy, Cat. No.: A5955);
- 50 mL polypropylene tubes (Sarstedt, Milan, Italy, Cat. No.: 62559001);
- 100 mm Petri dish (Sarstedt, Milan, Italy, Cat. No.: 833902);
- 35 mm Petri dish (Sarstedt, Milan, Italy, Cat. No.: 831800);
- Sterile scalpel (Swann-Morton, Sheffield, UK, Cat. No. 0506);
- Sterile scissors;
- Sterile tweezers;
- 500 mL plastic or glass bottles;
- Deionized water (DI-H_2_ 2O);
- Sterile water (Sigma-Aldrich, Milan, Italy; Cat. No.: W3500);
- Ethanol (Sigma-Aldrich, Milan, Italy; Cat. No.: 24105);
- Sodium dodecyl sulfate (SDS), (Sigma-Aldrich, Milan, Italy, Cat. No.: 74255);
- Triton X-100 (Sigma-Aldrich, Milan, Italy, Cat. No.: X100);
- Deoxycholate (SD) (Sigma-Aldrich, Milan, Italy, Cat. No.: D2510);
- Porcine gelatine (Sigma-Aldrich, Milan, Italy; Cat. No.: G1890);
- Dulbecco’s Modified Eagle Medium (DMEM; Thermo-Fisher, Milan, Italy, Cat. No.: 41966-029);
- Dulbecco’s Modified Eagle Medium/Nutrient Mixture F-12 (DMEM/F-12; Thermo-Fisher, Milan, Italy, Cat. No.: 11320-074);
- Antibiotic/antimycotic solution (Sigma-Aldrich, Milan, Italy; Cat. No.: A5955);
- Fetal Bovine Serum (FBS; Thermo-Fisher, Milan, Italy, Cat. No.: 10270-106);
- L-glutamine (Sigma-Aldrich, Milan, Italy; Cat. No.: G7513);
- Trypsin-EDTA (Sigma-Aldrich, Milan, Italy; Cat. No.: T3924).

### 2.2. Equipment

- Freezer at −80 °C (Argo-Lab, Milan, Italy; Cat. No.: SKO-D XL);
- Water bath (Chemimika, Pellezzano, Italy Cat. No.: GFL1003);
- Orbital shaker (ArgoLab, Carpi, Italy, Cat. No.: SKO-D XL);
- Inverted optical microscope (Nikon, Firenze, Italy, Cat. No.: TE200);
- CO_2_ incubator (Sanyo, Sanyo Electric Co., Osaka, Japan; Cat. No.: MCO-15AC).

### 2.3. Solutions

- 1% SDS: dissolve 5 g of SDS powder in 500 mL of DI-H_2_O;
- 1% Triton X-100: add 5 mL of Triton X-100 in 495 mL of DI-H_2_O;
- 2% SD: dissolve 10 g of SD in 500 mL of DI-H_2_O;
- Fibroblast culture medium: DMEM supplemented with 10% FBS, 1% L-glutamine, and a 1% antibiotic/antimycotic solution;
- Epithelial culture medium: DMEM/F12 supplemented with 10% FBS, 1% L-glutamine, and a 1% antibiotic/antimycotic solution;
- 0.1% porcine gelatine: dissolve 0.1g of porcine gelatin in 100 mL of distilled water. Autoclave and store at +4 °C.

## 3. Procedure

### 3.1. Porcine Intestine Collection

Collect porcine intestines at the local slaughterhouse (Tavazzano con Villavesco, Lodi, Italy) from freshly slaughtered animals, as described in Albl et al.’s protocol that ensures the generation of comparable and reproducible high-quality samples [45]. A maximum of 45 min can pass after animal sacrifice and organ collection. Lay the different intestinal segments in loops on a table. Identify and isolate around 1.5 m of jejunum (please note that the protocol described below can be applied to any other intestinal tracts). Transfer organs in sterile, cold PBS containing an antibiotic/antimycotic solution (5 mL/500 mL) and transport them to the laboratory (~60 min) using an ice container and maintain at a temperature of +4 °C. Upon arrival (around 2 h from animal sacrifice), process the samples immediately for decellularization (please see Section 3.2) and cell isolation (please see Section 3.3 and Section 3.4). Please note that samples cannot be stored.

### 3.2. Intestine Decellularization

**Figure 1:**
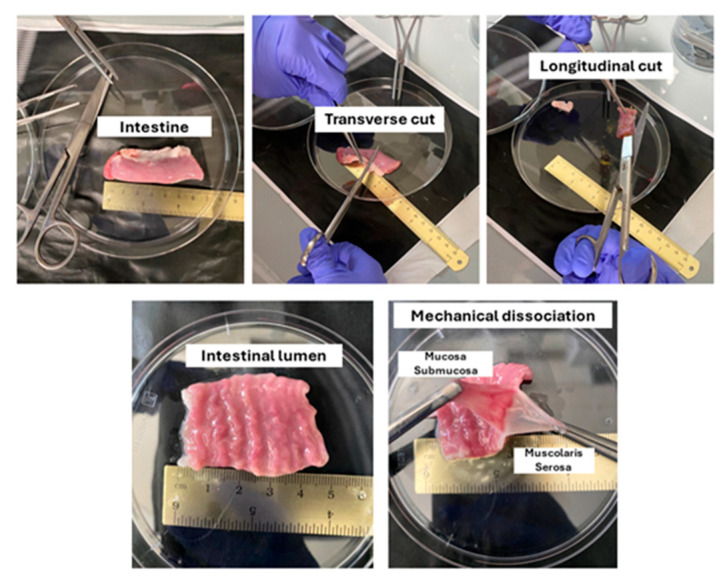
Sample preparation and intestinal mucosa isolation.

**Figure 2:**
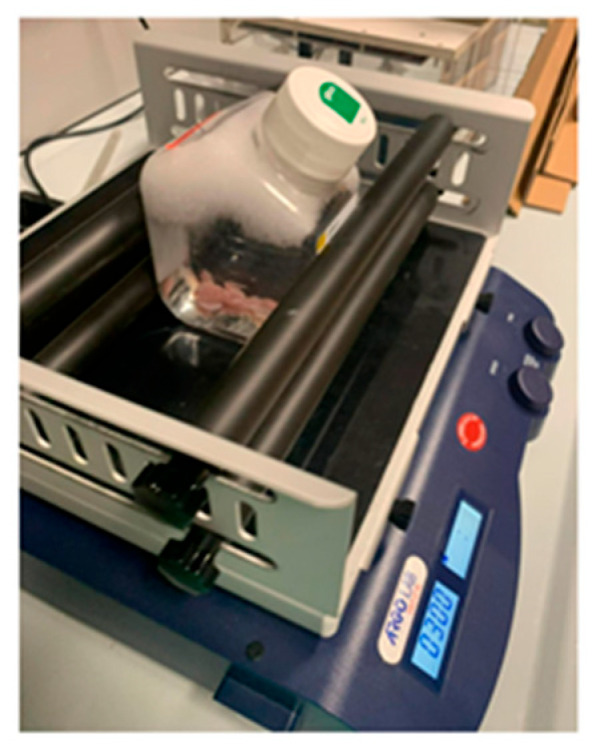
Incubation of intestinal fragments into a 500 mL bottle containing SDS using an orbital shaker.

Once at the laboratory, extensively wash the intestine in fresh PBS, transfer it to an empty 100 mm Petri dish, and cut it into pieces of 10 cm (length). Add tissues to a 50 mL tube and store at −80 °C for at least 24 h. Thaw the intestine at 37 °C for 45 min using a water bath. Transversally cut it into smaller fragments of around 5 cm (in length, Figure 1) and mechanically dissociate the intestinal mucosa and submucosal compartments from the tunica muscularis and serosa. Cut the tissues again into smaller fragments of around 2 cm and transfer them to a bottle containing 500 mL of a 1% SDS solution. Place the bottle onto an orbital shaker at 300 rpm and incubate for 12 h at room temperature (Figure 2). Remove the SDS solution from the bottle containing the intestinal fragments and wash them with 500 mL of DI-H_2_O for 12 h using an orbital shaker at 300 rpm at room temperature. At the end of the incubation time, remove the DI-H_2_O from the bottle, add 500 mL of the 1% Triton X-100 solution and incubate for 12 h at room temperature using an orbital shaker at 300 rpm. Remove the Triton X-100 solution from the bottle containing the fragments and wash them with 500 mL of DI-H_2_O for 12 h at room temperature using an orbital shaker at 300 rpm. Remove the DI-H_2_O from the bottle and incubate with 500 mL of a 2% SD solution for 12 h at room temperature using an orbital shaker at 300 rpm. Remove the deoxycholate and wash the intestinal fragments with DI-H_2_O three times. Finally, remove the last washing DI-H_2_O from the bottle and incubate the tissues in DI-H_2_O for 12 h at room temperature using an orbital shaker at 300 rpm. The obtained bio-scaffolds can be either directly used for histological analysis or sterilized for repopulation.

### 3.3. Porcine Intestinal Fibroblast Isolation

Collect porcine intestines at the local slaughterhouse immediately after animal slaughter. Transfer the intestines to sterile cold PBS supplemented with an antibiotic/antimycotic solution (5 mL/500 mL) and immediately transfer to the laboratory using an ice container, maintaining a temperature of +4 °C. Add 2 mL of 0.1% porcine gelatin to a 35 mm culture dish and incubate for 45 min at room temperature. Wash the tissue biopsies in sterile fresh PBS. Cut the intestine into pieces of around 5 cm and mechanically dissociate the intestinal mucosa and tunica muscularis from the connective layer. Transfer the tissue to a new sterile Petri dish and add a fresh fibroblast culture medium. Cut the tissue into small fragments of approximately 2 mm^3^ using a surgical scalpel. Eliminate porcine gelatin from the 35 mm Petri dish and add 5–6 fragments, letting the connective attach to the gelatin in the culture support. Then, add 15 µL of a fresh fibroblast culture medium and incubate at 37 °C. Add fibroblast culture medium drops (~20–50 µL) on all the following days. At day 6, add 1 mL of the fibroblast culture medium and maintain in culture until confluence. When confluency is reached, detach cells from the culture support and transfer to a new one (see Section 3.5).

### 3.4. Porcine Intestinal Epithelial Cell Isolation

Collect porcine intestines at the local slaughterhouse immediately after animal slaughter. Transfer the intestines to sterile cold PBS supplemented with an antibiotic/antimycotic solution (5 mL/500 mL) and immediately transfer to the laboratory using an ice container. Prepare 2 mL of 0.1% porcine gelatin in a 35 mm culture dish and incubate for 45 min at room temperature. Wash the intestines extensively in fresh PBS. Cut the intestines into fragments of around 5 cm and mechanically dissociate the intestinal mucosa from the submucosa and tunica muscularis. Transfer the tissue to a new sterile Petri dish and add a fresh epithelial culture medium. Cut the tissue into small fragments of approximately 2 mm^3^ using a surgical scalpel. Eliminate porcine gelatin from the 35 mm Petri dish and add 5–6 fragments, letting the epithelial layer attach to the gelatin in the culture support. Add 15 µL of a fresh epithelial culture medium and incubate at 37 °C. Add epithelial culture medium drops (~20–50 µL) on all the following days. At day 5, add 1 mL of the epithelial culture medium and maintain in culture until confluence. When confluency is reached, detach cells from the culture support and transfer to a new one (see Section 3.5).

### 3.5. Porcine Intestinal Fibroblast and Epithelial Cell Propagation and Maintenance

Culture intestinal fibroblasts and epithelial cells in an incubator (5% CO_2_) at 37 °C and check their morphology and growth daily. When cells reach 80% confluency, remove the culture media and carefully wash cells using PBS with a 1% antibiotic/antimycotic solution thrice. After the last wash in PBS, incubate the cell monolayers in a trypsin-EDTA solution at 37 °C for around 5 min and check for cell detachment from the bottom of the tissue culture dishes. Dilute the cell suspension in a fibroblast or epithelial culture medium containing FBS to block trypsin activity. Dissociate cells by gently pipetting many times. Place the cell suspensions in a new 15 mL centrifuge polystyrene tube and centrifuge at 300× *g* for 5 min. Eliminate the cell supernatants and add the appropriate fresh culture medium. Resuspend the cell pellets by gently pipetting and transferring them to a new culture support. Maintain in the incubator (5% CO_2_) at 37 °C and change the medium every 2–3 days. Keep the passage ratio between 1:2 and 1:4 based on the cell growth rate.

### 3.6. Repopulation of Intestinal Bio-Scaffolds with Intestinal Fibroblasts and Epithelial Cells

Remove the last wash in DI-H_2_O from the bottle containing the decellularized bio-scaffolds and, under a sterile hood, transfer the fragments into ethanol (70%) supplemented with a 2% antibiotic/antimycotic solution. Incubate for 30 min at room temperature, using an orbital shaker at 300 rpm. Then, remove the ethanol (70%) and transfer the fragments to a 50 mL sterile tube containing warm PBS supplemented with the 2% antibiotic/antimycotic solution. Incubate for 1 h at room temperature using an orbital shaker at 300 rpm. Remove the PBS and cut the fragments into smaller pieces of 3 × 3 mm^2^. Transfer the fragments to warm DMEM containing a 2% antibiotic/antimycotic solution and incubate for 1 h at 37 °C and 5% CO_2_. Aspirate the fibroblast culture medium from the cells, wash the intestinal fibroblasts with PBS containing a 1% antibiotic/antimycotic solution, and detach them from the culture support using a trypsin-EDTA solution at 37 °C. Add a fresh medium to the detached cells and transfer to a 15 mL centrifuge polystyrene tube. Calculate the cell number using a counting chamber under an inverted optical microscope. Determinate the medium volume required to resuspend cells at a 1 × 10^6^ cells/cm^2^ concentration. After cell centrifugation at 300× *g* for 5 min, remove the supernatant and add the previously calculated volume of fibroblast culture medium. Repopulate the intestinal bio-scaffold by pipetting fibroblasts on top of it and carefully transfer them into a CO_2_ incubator. Culture the cells for 14 days and change the culture medium every 48 h. Eliminate the intestinal epithelial medium from the culture dish and, after three washes in PBS, detach the cell monolayer using the trypsin-EDTA solution at 37 °C. Resuspend the detached cells in a fresh epithelial culture medium and centrifuge the cell suspension using a 15 mL polystyrene tube. Calculate the cell number using a counting chamber under an inverted optical microscope. Determinate the medium volume required to resuspend cells at a 1 × 10^6^ cells/cm^2^ concentration. After cell centrifugation at 300× *g* for 5 min, remove the supernatant and add the previously calculated volume of the fibroblast culture medium. Finally, add epithelial cells to the fibroblast-repopulated bio-scaffold and carefully transfer them into the CO_2_ incubator. Culture the cells for 14 days.

## 4. Expected Results

**Figure 3:**
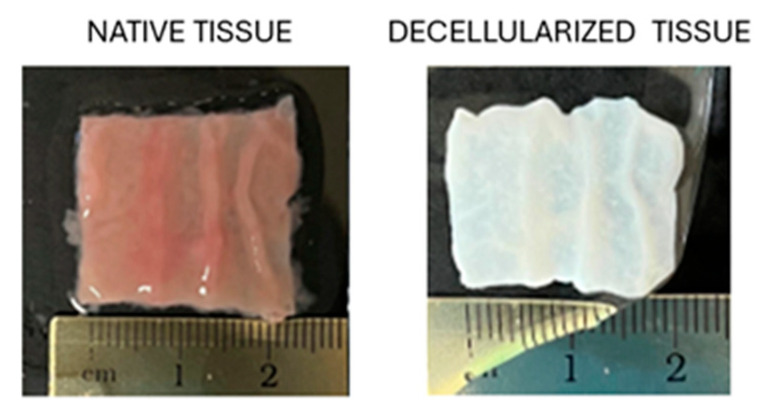
Porcine intestine before (native tissue) and after decellularization (decellularized tissue).

At the end of the decellularization process, a macroscopic evaluation shows that the color of intestinal bio-scaffolds turns from red to white, suggesting the successful removal of the cell component. In addition, the generated bio-scaffolds maintain the original tissue shape with visible intestinal folds and without any deformation (Figure 3).

**Figure 4:**
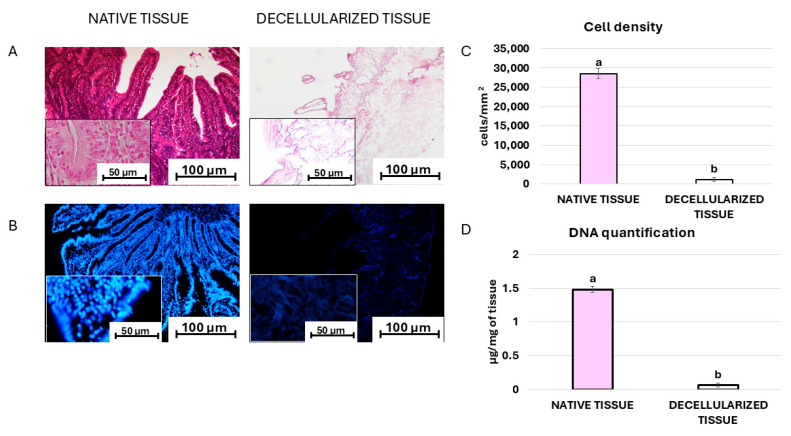
(**A**) Hematoxylin/eosin and (**B**) DAPI staining of native and decellularized tissue (scale bars 100 µm and 50 µm). (**C**) Cell density analysis of native (pink bar) and decellularized tissue (white bar). (**D**) DNA quantification analysis of native (pink bar) and decellularized (white bar) tissue. ^a,b^ Different superscripts indicate significant differences (*p* < 0.05).

Cell removal is confirmed by microscopic evaluations, which can be carried out using hematoxylin/eosin and DAPI staining following the protocol previously described [5,18]. In particular, the results obtained with hematoxylin/eosin demonstrate the absence of basophilic staining in the decellularized bio-scaffolds. In contrast, both the basophilic and eosinophilic staining are visible in the native tissue (Figure 4A). In line with this, DAPI staining and cell density analysis indicate a statistically significant lower number of nuclei in the decellularized intestines compared to the native ones (Figure 4B,C). DNA quantification supports these observations, showing a significant reduction in the DNA content in decellularized organs compared to native tissue (Figure 4D).

**Figure 5:**
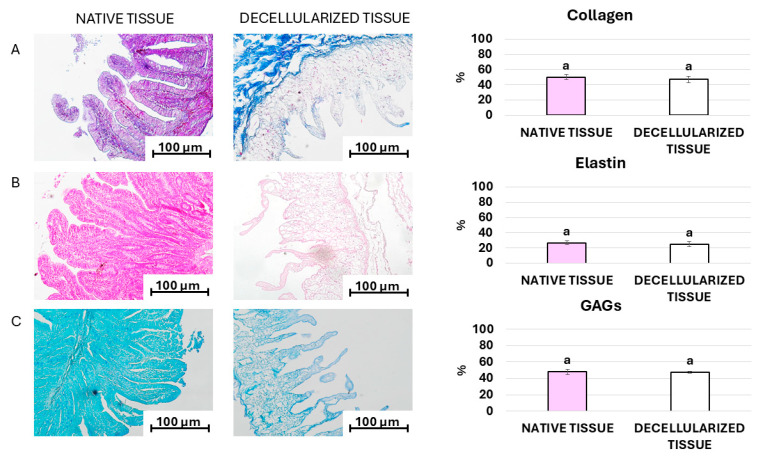
(**A**) Crossmon’s trichrome staining for collagen fibers, (**B**) Gomori’s aldehyde-fuchsin paraldehyde for elastic fibers, (**C**) alcian blue staining for glycosaminoglycans (GAGs) staining (scale bars 100 µm) and stereological quantifications of native (pink bar) and decellularized tissue (white bar). ^a^ Same superscript indicates no significant differences (*p* > 0.05).

Histochemical evaluations and stereological analyses indicate that the protocol developed maintains statistically comparable amounts of the main ECM components compared to the native tissue (Figure 5). In particular, Crossmon’s trichrome staining shows the preservation of collagen fibers after the decellularization process and the maintenance of standard localization in the lamina propria of the tissue (Figure 5A). In addition, stereological analyses confirm a statistically comparable amount of collagen fibers between the decellularized bio-scaffolds and the control tissue (native tissue, Figure 5A). Similarly, Gomori’s aldehyde fuchsin, as well as alcian blue staining and its stereological analysis, demonstrate the preservation of intact elastin fibers (Figure 5B) and glycosaminoglycans (GAGs; Figure 5C) at the end of the decellularization process, respectively. 

**Figure 6:**
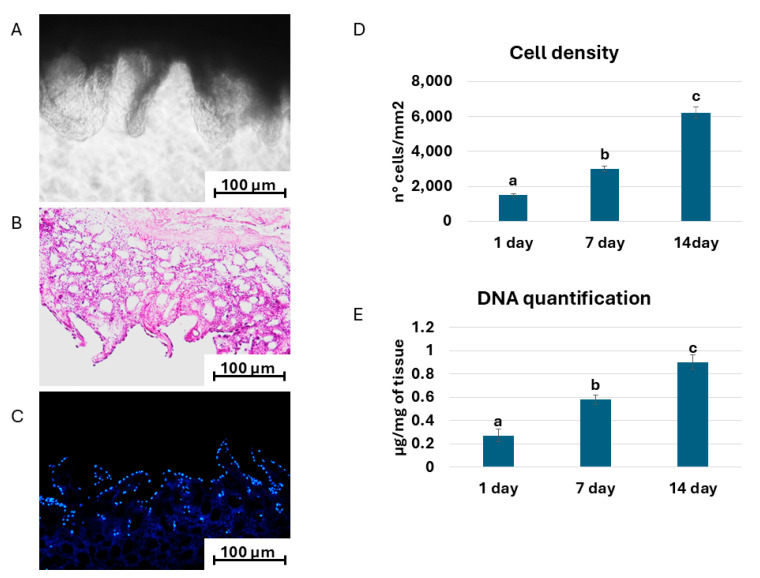
(**A**) Representative image of repopulated intestinal bio-scaffold (scale bar 100 µm); (**B**) hematoxylin/eosin staining (scale bar 100 µm); and (**C**) DAPI staining (scale bar 100 µm). (**D**) Cell density analysis after 1, 7, and 14 days of culture. (**E**) DNA quantification analysis of repopulated bio-scaffolds (blue bars) after 1, 7, and 14 days of culture. ^a,b,c^ Different superscripts indicate significant differences (*p* < 0.05).

When intestinal fibroblasts and epithelial cells are seeded onto the decellularized intestinal bio-scaffolds, they rapidly migrate into the bio-scaffolds, adhering and colonizing the ECM within 24 h, ruling out any cytotoxic effects possibly exerted by the decellularized intestine (Figure 6A). These observations are also confirmed by hematoxylin/eosin staining (Figure 6B), which shows the presence of basophilic cells. In parallel, DAPI staining (Figure 6C) and cell density analysis (Figure 6D) confirm a steady increase in cell numbers over the culture period, indicating the bio-scaffold’s ability to encourage cell homing. In agreement with these data, DNA quantification analysis shows the presence of DNA in the repopulated bio-scaffolds, with a significant increment during the entire length of the culture period (Figure 6E).

## 5. Conclusions

The decellularization protocol described here combines the use of mechanical forces and chemical reagents to successfully generate intestinal bio-scaffolds that preserve the shape, architecture, and ECM composition. Thanks to these features, the obtained 3D biological supports repopulated with intestinal fibroblasts and epithelial cells allow for the creation of highly predictive 3D platforms that mimic the complex architecture and functions of the intestinal barrier and can find useful applications for nutritional, toxicological, and pharmaceutical studies.

## Data Availability

The data presented in this study are available on request from the corresponding author.
